# Ileal Digestibility of Crude Protein and Amino Acids in Bakery and Biscuit Byproducts Fed to Growing Pigs

**DOI:** 10.3390/ani16193087

**Published:** 2026-09-30

**Authors:** Chaibin Lim, Hyunjung Jung, Joeun Kim, Yejin Min, Yohan Choi, Hongjun Kim, Sungwoo Park, Hyunju Park, Jinyoung Jeong, Yongdae Jeong

**Affiliations:** 1 Swine Science Division, National Institute of Animal Science, Rural Development Administration, Cheonan 31000, Republic of Korea; blight112@gmail.com (C.L.);; 2 Precision Animal Nutrition Division, National Institute of Animal Science, Rural Development Administration, Wanju 55365, Republic of Korea

**Keywords:** amino acid digestibility, standardized ileal digestibility, bakery byproducts, growing pig, alternative feed ingredient

## Abstract

This study evaluated the apparent ileal digestibility (AID) and standardized ileal digestibility (SID) of crude protein (CP) and amino acids (AAs) in three bakery and biscuit byproducts fed to growing pigs. Five crossbred barrows fitted with a T-cannula at the distal ileum were allotted to a 5 × 5 Latin square design. The AID of CP was higher in the plain bread meal (PB) and sweet bread meal (SB) than in corn (CN) and biscuit meal (BC) (*p* < 0.05), while the AID of most indispensable AAs was higher in CN and PB than in BC (*p* < 0.05), reflecting the dietary CP and total AA concentrations. After correction for basal endogenous AA losses, the SID of CP separated the ingredients more clearly, with PB and SB higher than CN, and CN higher than BC (*p* < 0.05). The SID of lysine, methionine, threonine, and tryptophan did not differ among the four ingredients, indicating that the digestible supply of these limiting AAs was maintained when corn was replaced with these ingredients. These SID values provide ingredient-level information for incorporating bakery and biscuit byproducts into growing pig diets as alternative feed ingredients alongside corn.

## Simple Summary

Unsold or surplus bread, cake, and biscuit products from food manufacturing are often discarded, even though they still contain valuable nutrients such as energy and protein. Using these bakery and biscuit byproducts as animal feed could reduce food waste and lower the reliance on cereal grains also used for human food. Because pig growth depends on digestible amino acids, the building blocks of protein, the value of a feed ingredient depends largely on how well its amino acids are digested. In this study, we evaluated the digestibility of protein and amino acids in three bakery and biscuit byproducts in growing pigs, with corn as a reference. The byproducts supplied digestible amino acids comparable to corn, indicating that they can replace part of the corn in pig diets without reducing the supply of the most limiting amino acids. These findings provide nutritionists with reference values for incorporating bakery and biscuit byproducts into growing pig diets, supporting more sustainable and cost-effective pork production.

## 1. Introduction

Diversification of feed ingredients is a recurring priority in monogastric animal nutrition to stabilize compound feed costs and improve the sustainability of livestock production systems [1]. Pork production places substantial demand on global cereal supplies, with pig feed accounting for a major share of cereals diverted from human consumption to livestock [2]. Climate change and the ongoing increase in global food demand further intensify competition between feed and food production systems [3]. A practical strategy to mitigate this competition is the recovery of food byproducts generated during human food manufacturing [4]. Bakery materials, in particular, have been characterized as alternative feed ingredients for monogastric animals [5]. Unsold or surplus bakery and biscuit products that would otherwise be discarded as food waste can be recovered and used as feed ingredients [6]. Bakery products are produced in large volumes, contain readily available starch and lipid fractions, and are amenable to drying and milling for incorporation into compound feed [7]. Bakery and biscuit materials enter the byproduct stream as unsold, surplus, or off-specification product, and are converted into feed ingredients through drying and grinding without further refining. The composition of the resulting meal therefore reflects that of the originating product stream. Plain bread, cream-filled sweet bread, and biscuit constitute three streams that are separately collected and marketed, and separate evaluation of each stream is a prerequisite for assigning ingredient-specific values in diet formulation.

Diet formulation for growing pigs is increasingly based on standardized digestible amino acid (AA) values rather than total or apparent values. In low-AA diets, basal endogenous losses (BEL) constitute a proportionally larger fraction of total ileal outflow [8]. Accordingly, the apparent ileal digestibility (AID) of an AA reflects not only the inherent digestibility of the feed ingredient but also the concentration of that AA in the assay diet. Adjusting AID for these BELs yields the standardized ileal digestibility (SID), which more accurately reflects the ingredient itself and is additive when multiple ingredients are combined in a compound diet [9]. Modern swine feed databases therefore depend on ingredient-level SID values to accurately predict the dietary supply of digestible AAs.

The SID of AAs varies more widely among bakery meal sources than among conventional feed ingredients [10], reflecting differences in raw materials, baking conditions, and drying intensity. Surveys of bakery meal have reported wide variation in chemical composition and energy concentrations across processing sources [11]. Feeding trials have demonstrated that bakery products can replace cereal grains in growing pig diets at moderate inclusion levels without compromising growth performance [12]. A recent evaluation of bakery and biscuit byproducts in growing pigs reported digestible and metabolizable energy concentrations exceeding those of corn, along with nitrogen retention comparable to that of a corn-based control [13]. Available data on the ileal AA digestibility of these materials have been obtained largely from bakery meal, which is produced by blending bread, cookie, and cracker fractions, and predominantly from North American processing operations. Bread and biscuit products that are manufactured and marketed as separate commodities have not been evaluated individually, and ingredient-level digestibility values for such materials are therefore lacking. The present study was conducted to determine the AID and SID of CP and AAs in three bakery and biscuit byproducts fed to growing pigs.

## 2. Materials and Methods

The protocol for this study was approved by the Institutional Animal Care and Use Committee of the National Institute of Animal Science (Approval No. NIAS2024-0023).

### 2.1. Experimental Facilities

The trial was conducted at the experimental swine unit of the National Institute of Animal Science (Cheonan, Republic of Korea). Each pig was housed individually in a stainless steel metabolism crate (45 × 55 × 45 cm) equipped with a self-feeder and a nipple drinker, with free access to water throughout the study. Room temperature and relative humidity were maintained at 22 °C ± 2 °C and 60% ± 5%, respectively, using an automated climate-control system under a 12 h light/dark cycle.

### 2.2. Animals, Experimental Design, and Diets

Corn and the bakery- and biscuit-derived test ingredients were obtained from two commercial suppliers, DH Vital Feed (Pyeongtaek, Republic of Korea) and Hanjungss (Seoul, Republic of Korea). Plain bread meal (PB) was prepared from dried white bread, sweet bread meal (SB) from cream-filled bakery products, and biscuit meal (BC) from a mixture of cookies and crackers. Ingredient batches were ground at the National Institute of Animal Science and analyzed before diet formulation. The detailed chemical composition of the test ingredients was previously reported by Lee et al. [13] and is summarized in Table 1. Five crossbred barrows [(Landrace × Yorkshire) × Duroc] (initial body weight 17.22 ± 1.17 kg) were fitted with a T-cannula at the terminal ileum following the surgical procedure described by Stein et al. [14]. The pigs were assigned to five dietary treatments in a 5 × 5 Latin square design (Table 2): a corn-based control diet (CND); three test diets in which PB (PBD), SB (SBD), or BC (BCD) served as the sole source of CP and AA; and a nitrogen-free diet used to estimate BELs of AAs. Each diet was supplemented with vitamins and minerals to satisfy or surpass the nutrient requirements for growing pigs recommended by the National Research Council [15]. Chromic oxide, an indigestible marker, was included in all diets at 0.40% [16]. Body weight was measured at the beginning of each period (day 0), and the daily feed allowance was calculated as three times the maintenance metabolizable energy requirement (197 kcal ME/kg body weight^0.60^) [15]. The daily ration was offered once each morning at 08:00, and feed refusals were collected and weighed to determine actual feed intake.

### 2.3. Sample Collection and Chemical Analysis

Each period began with a 5-day dietary adaptation phase, followed by ileal digesta collection over two consecutive days for 8 h each day. A polyethylene bag was attached to the open end of the T-cannula with cable ties, and digesta were collected continuously as they flowed into the bag. The bag was replaced at 30 min intervals. Each collected sample was immediately stored at −20 °C. After completion of the collection period, digesta from each pig within each period were pooled across all bags and collection days, and a representative subsample was retained for subsequent analyses. After freeze-drying and grinding, the pooled subsamples were subjected to chemical analyses. Dry matter and CP contents were determined according to the Association of Official Analytical Chemists methods 930.15 and 990.03, respectively [17]. The AA composition of the diets and digesta was analyzed using an L-8800 AA analyzer (Hitachi High-Technologies America, Pleasanton, CA, USA), with sample preparation and chromatographic separation performed as described by Llames and Fontaine [18]. Chromium concentrations in the diets and ileal digesta were determined using the colorimetric method of Fenton and Fenton [16]. The AID, basal endogenous AA losses, and SID of CP and individual AAs were calculated according to the equations of Stein et al. [19]. The calculations were based on the chromium and AA concentrations in the diets and digesta, with basal endogenous AA losses estimated from pigs fed the nitrogen-free diet. Because each test ingredient was the sole dietary source of CP and AAs in its respective experimental diet, the AID and SID values calculated for each diet represent the digestibility of CP and AAs in the corresponding ingredient. Concentrations of standardized ileal digestible AA in each ingredient were subsequently obtained by multiplying the SID coefficient determined for the corresponding diet by the analyzed AA concentration of that ingredient, and are expressed on an as-fed basis.

### 2.4. Statistical Analysis

Data were analyzed using the MIXED procedure of SAS (version 9.4; SAS Institute Inc., Cary, NC, USA) as a 5 × 5 Latin square with five pigs and five periods. Dietary treatment was included as a fixed effect, and pig and period as random effects with a variance-components covariance structure; denominator degrees of freedom were determined by the containment method. Treatment means were separated using Tukey’s adjustment within each response variable, without further correction for multiplicity across the CP and AA panel. Significance was declared at *p* < 0.05.

## 3. Results

### 3.1. Apparent Ileal Digestibility

The effects of dietary bakery and biscuit byproducts on the AID of CP and AAs are shown in Table 3. The AID of CP was higher in PB and SB than in CN and BC (*p* < 0.05). The AID of arginine, histidine, methionine, phenylalanine, threonine, and total indispensable AAs was higher in CN and PB than in BC (*p* < 0.05). A similar pattern was observed for alanine, aspartic acid, cystine, serine, and tyrosine, as well as for total dispensable AAs and total AAs (*p* < 0.05). Additionally, the AID of isoleucine was higher in CN than in BC (*p* < 0.05), whereas the AID of glutamic acid was lower in SB than in the other treatments (*p* < 0.05). No significant differences among treatments were detected for the remaining AAs.

### 3.2. Standardized Ileal Digestibility

The effects of dietary bakery and biscuit byproducts on the SID of CP and AAs are presented in Table 4. The SID of CP was higher in PB and SB compared with CN (*p* < 0.05), and higher in CN than in BC (*p* < 0.05). The SID of histidine was higher in CN and PB than in BC (*p* < 0.05). Leucine showed a higher SID in BC than in CN (*p* < 0.05). Among the dispensable AAs, the SID of cystine and tyrosine was higher in CN compared with all other treatments (*p* < 0.05). The SID of glutamic acid was higher in BC than in SB (*p* < 0.05). No significant differences were observed among treatments for the remaining AAs.

### 3.3. Concentrations of Standardized Ileal Digestible Amino Acids

The concentrations of standardized ileal digestible AAs in CN, PB, SB, and BC are presented in Table 5. Standardized ileal digestible lysine was 0.18, 0.45, 0.36, and 0.14% in CN, PB, SB, and BC, respectively, and the corresponding values for threonine were 0.22, 0.39, 0.32, and 0.15%.

## 4. Discussion

Bakery- and biscuit-derived materials are receiving increasing attention as alternative sources of energy and protein in swine diets, although their composition varies with raw material source, drying intensity, and downstream processing [11]. Indeed, the variability in the SID of AAs among bakery meal sources has been reported to exceed that typically observed in conventional feed ingredients [10]. Reliable data on the ileal AA digestibility of bakery and biscuit byproducts produced outside North American supply chains remain limited. In the present study, CN, PB, SB, and BC were each included at the same dietary level in separate assay diets. Because each ingredient was the sole source of CP and AAs in its assay diet, the resulting values represent the digestibility of CP and AAs in the ingredient. This approach is consistent with the direct-method framework commonly used to determine the ileal AA digestibility of swine feed ingredients [20].

The AID of CP was higher in PB and SB than in CN and BC, and the AID of most indispensable AAs was higher in CN and PB than in BC, with SB showing intermediate values that did not differ significantly from those of either group. AID values have been shown to decrease as dietary CP and AA concentrations decline [21]. The lower CP content of BC than of PB and SB was reflected in the lower analyzed dietary CP and total AA concentrations in BCD. This decrease occurs because basal endogenous AA losses constitute a relatively large fraction of total ileal AA outflow when the dietary AA supply is reduced [22]. The relationship between dietary AA concentration and AID follows a curvilinear pattern. AAs that contribute substantially to basal endogenous AA losses exhibit steeper slopes, whereas those with smaller endogenous contributions exhibit shallower slopes. This differential sensitivity has been confirmed across a range of feed ingredients and inclusion levels [23]. The AID ranking observed in the present study is therefore more consistent with the dietary AA density of each diet than with the intrinsic digestibility of the test ingredient.

The neutral detergent fiber concentration of PB exceeded that of the other three ingredients, yet the AID of AAs in PB did not exceed the corresponding values in CN for any AA. At the inclusion level applied in the present study, the resulting difference in calculated dietary neutral detergent fiber between PBD (8.12%) and CND (4.65%) was modest relative to the difference at the ingredient level. The fiber fraction of plain bread meal originates from wheat flour and bran, in which the nonstarch polysaccharides are less lignified than those of many cereal co-products [24]. Detergent-fiber procedures applied to baked, starch-rich matrices may further retain resistant starch within the residue unless starch is fully removed during the analysis [25], such that the tabulated neutral detergent fiber concentration may overstate the structural fiber content of bakery materials.

SID values were obtained by correcting AID for basal endogenous AA losses determined in pigs fed the nitrogen-free diet [26]. The SID of CP was significantly higher in PB and SB than in CN and lowest in BC. Thus, SID provided a clearer separation among the four ingredients than AID. Conversely, BEL correction reduced or eliminated most of the differences observed in indispensable AA digestibility at the AID level. The decrease in AID observed for BC was largely resolved after BEL correction. The corrected values therefore more closely reflect the digestibility of AAs on a per-unit basis than the corresponding AID values do, and provide a more appropriate basis for evaluating bakery and biscuit byproducts as feed ingredients.

Although the SID of CP differed among ingredients, the SID of total indispensable AAs, total dispensable AAs, and total AAs did not (Table 4). Among individual AAs, the increase from AID to SID was largest for leucine in BC (35.0 percentage points, compared with 11.7 in CN). BC contained the lowest leucine concentration of the four test ingredients, and because the BEL correction is inversely proportional to the dietary AA concentration, the correction applied to leucine in BCD was disproportionately large. The nitrogen-free method can produce inflated SID values when the dietary concentration of an AA is very low [27]; the leucine pattern observed here may therefore reflect this methodological effect rather than superior leucine digestibility in BC.

A single set of basal endogenous losses, determined from pigs fed the nitrogen-free diet and expressed per kilogram of DM intake, was applied to all four diets. The basal endogenous losses of leucine and valine determined here were comparable to published values for growing pigs [9], whereas those of CP, arginine, isoleucine, lysine, and threonine were lower and those of methionine and phenylalanine were higher. Estimates of basal endogenous losses vary widely among studies [28], and part of this variation arises from the method itself: feeding a nitrogen-free diet alters endogenous protein secretion and body protein metabolism [29], and the carbohydrate composition of the nitrogen-free diet affects ileal endogenous AA flow [22]. The very low values obtained for histidine and tryptophan most likely reflect concentrations of these AAs in the ileal digesta of pigs fed the nitrogen-free diet that were close to the limit of quantification. Because the losses of most indispensable AAs were lower than published estimates, the correction from AID to SID was correspondingly smaller, and the SID values reported here, particularly those of histidine and tryptophan, should be regarded as conservative estimates. Despite this variation, nitrogen-free correction has long been the standard approach for determining SID in ileal digestibility studies in pigs [19]. The SID values obtained using this approach remain the operational basis of most modern feed digestibility databases for swine diet formulation [15]. Unlike AID values, SID values are additive when multiple ingredients are combined in mixed diets [30]. This additivity allows the digestibility of a complete diet to be predicted from the SID values of its individual ingredients. Within the limitations noted above, the SID values determined here can be applied to the formulation of growing pig diets containing bakery and biscuit byproducts.

After BEL correction, the SID values of several AAs differed significantly among the four ingredients, including cystine, tyrosine, histidine, and glutamic acid. The interpretation of these localized differences requires caution. Chemical markers of heat-induced AA modification were not measured in the present study, and the nitrogen-free method does not distinguish ingredient-specific endogenous losses from basal endogenous losses [27]. Therefore, the localized SID differences may reflect intrinsic differences in ingredient digestibility combined with ingredient-specific endogenous losses that are not accounted for by this method. Heat processing during bakery and biscuit production may also contribute to these differences. Heat treatment of protein ingredients has been shown to reduce the ileal digestibility of indispensable AAs in growing pigs, with sulfur-containing AAs being particularly susceptible to thermal damage [31]. Accordingly, these localized differences should not be interpreted directly as evidence of ingredient-specific protein quality. Instead, the overall nutritional value of the three byproducts is better assessed by jointly considering CP, the SID of limiting AAs, and energy density.

Synthesis of the AID, SID, and chemical composition data positions the three bakery and biscuit byproducts primarily as energy-dense feed ingredients that also provide useful amounts of standardized digestible AAs to growing pig diets. The conventional classification of feed ingredients as either protein or energy sources offers limited value for these heterogeneous byproducts because the protein and energy fractions of bakery materials vary with the underlying raw materials. The nutritional value of each ingredient is therefore best evaluated using two complementary dimensions: digestible energy density and standardized digestible AA contribution per unit weight.

The analyzed CP concentrations of PB and SB exceeded those of corn, and both ingredients may therefore contribute digestible AAs beyond what corn alone supplies in a mixed diet. The SID of lysine, methionine, threonine, and tryptophan did not differ among the four ingredients, indicating that the digestible supply of these limiting AAs was maintained when corn was replaced with PB, SB, or BC at the inclusion rate examined here. Cystine followed a different pattern, with a lower SID in all three byproducts than in CN. Assigning this contrast to a specific chemical mechanism is not possible from the present data, because furosine and reactive lysine were not determined. Assigning these contrasts to a specific chemical mechanism is not possible from the present data, because furosine and reactive lysine were not determined. Lysine is the AA blocked directly by Maillard reactions in heat-processed ingredients, and reactive lysine serves as the analytical index of the extent of this modification [32]. The absence of a lysine response in the present study therefore provides no evidence of extensive Maillard-type modification in these ingredients. Cystine, however, is susceptible to oxidative loss during thermal processing through pathways independent of Maillard chemistry [33], which may account for the lower SID of cystine in the byproducts. Determination of furosine and reactive lysine would be required to establish whether processing-related modification contributes to the cystine contrasts observed here. Within the limits of the present dataset, PB and SB may be suitable for partial replacement of corn as complementary contributors of digestible AAs in corn-based growing pig diets.

Among the three byproducts, BC occupies a distinct nutritional position. The CP concentration of BC was comparable to that of corn, and its total AA concentration was the lowest among the three byproducts, though comparable to that of corn. However, after BEL correction, the SID of the limiting AAs in BC did not differ significantly from those of corn. The digestibility of AAs in BC on a per-unit basis is therefore more comparable to that of corn than the AID rankings of BC would suggest. BC also contained the highest ether extract and gross energy concentrations among the four ingredients, further supporting its classification as an energy-rich feed ingredient. In practical diet formulation, BC is best evaluated and priced according to its digestible energy density and standardized digestible AA contribution per unit weight. AID-based rankings derived from a single high inclusion level are not an appropriate basis for evaluating BC. Therefore, BC is best regarded as an energy-rich ingredient comparable to corn that also provides useful amounts of digestible AAs, rather than as an intrinsically low-quality protein source.

The corn-based control diet provides the reference against which the coefficients determined for the three byproducts should be read. The SID of lysine, methionine, threonine, and tryptophan in CN was 84.4%, 89.2%, 81.1%, and 68.7%, respectively, whereas the values tabulated for corn by the National Research Council are 74%, 83%, 77%, and 80%, each accompanied by a standard deviation exceeding nine percentage points [15]. The SID of lysine, methionine, and threonine determined for corn fell within one standard deviation of the corresponding tabulated means, and that of tryptophan within 1.2 standard deviations, reflecting the substantial variation among the corn samples from which the tabulated values were derived. The corn control values obtained here are therefore consistent with the published range for corn, which supports the validity of the assay conditions applied in the present study. An earlier evaluation reported that the SID of most AAs in bakery meal was lower than in corn [20], whereas the SID of most AAs in PB, SB, and BC did not differ from that in CN in the present study. Bakery meal in that earlier work was a commingled commodity stream, and the SID of AAs has since been shown to differ markedly among bakery meal sources [10]. The bread and biscuit streams evaluated here were collected and processed separately rather than commingled, and the digestible AA supply they provided was accordingly comparable to that of corn.

The published SID values of CP and indispensable AAs in bakery meals span a wide range across processing sources and product streams. In the present study, the SID of lysine in PB, SB, and BC fell within the upper portion of the range reported for 11 U.S. bakery meal sources, in which the mean SID of lysine was 71.6% [10]. Likewise, the SID values of bakery meals reported for young growing pigs with body weights comparable to those in the present study fell within a similar range [34]. Bakery meals have also been characterized as energy-rich ingredients with digestible and metabolizable energy concentrations comparable to or exceeding those of corn [11]. A recent evaluation of bakery and biscuit byproducts in growing pigs likewise reported digestible and metabolizable energy concentrations higher than those of corn, together with nitrogen retention similar to that of a corn-based control [13]. Collectively, the SID values obtained in the present study and those reported previously support the inclusion of PB, SB, and BC in growing pig diet formulations.

Ileal digestibility assays in cannulated pigs necessarily involve a limited number of animals, and the values obtained from such studies should be interpreted as informative estimates of ingredient digestibility. Nonsignificant treatment differences should therefore be regarded as an absence of detectable differences, not as evidence of equivalence. Source-level variability in bakery products is well-documented across processing sources [10], and the SID coefficients reported here should be applied to other batches with appropriate caution and, ideally, in conjunction with batch-specific analyses of CP and AA composition. Comparable variability has also been reported for other heat-processed feed byproducts in growing pigs, with raw material source and processing conditions identified as the principal contributors [35]. Direct chemical markers of heat-induced AA modification were not measured in the present study. Therefore, the proposed effects of thermal processing remain hypothetical and warrant further analytical investigation. Growth performance and nutrient utilization studies conducted at commercially relevant inclusion levels are also needed before the SID values determined here can be translated into expected responses in growing pigs [36]. Despite these limitations, the present study provides ingredient-level standardized digestibility coefficients of AAs for PB, SB, and BC to support diet formulation in growing pigs.

## 5. Conclusions

The present study evaluated the AID and SID of CP and AAs in PB, SB, and BC relative to a corn-based control in growing pigs. The SID of lysine, methionine, threonine, and tryptophan did not differ significantly among the four ingredients, supporting the inclusion of these three byproducts in corn-based diets for growing pigs. PB and SB, which contained higher CP concentrations than corn, can provide additional digestible AAs in mixed diets, whereas BC can serve as an energy-dense feed ingredient with usable digestible AA value. The standardized digestibility coefficients of AAs determined in the present study provide the ingredient-level information required for the accurate incorporation of bakery and biscuit byproducts into growing pig diet formulations, supporting their practical use as alternative feed ingredients alongside corn.

## Figures and Tables

**Table 1:** Composition of feed ingredient byproducts.

Items, %	CN	PB	SB	BC
Dry matter	84.3	87.5	88.1	88.9
Gross energy, kcal/kg	3879	4001	4231	4829
Crude protein	7.97	12.10	12.30	8.01
Crude ash	1.14	3.59	2.31	4.06
Ether extract	3.34	4.02	6.62	10.62
Neutral detergent fiber	9.30	16.24	12.24	5.50
Acid detergent fiber	3.10	5.93	7.62	2.20
Indispensable amino acids				
Arginine	0.35	0.70	0.58	0.31
Histidine	0.22	0.34	0.28	0.14
Isoleucine	0.25	0.58	0.48	0.23
Leucine	0.99	1.07	0.89	0.47
Lysine	0.21	0.58	0.48	0.18
Methionine	0.16	0.22	0.18	0.11
Phenylalanine	0.35	0.68	0.57	0.32
Threonine	0.27	0.50	0.42	0.19
Tryptophan	0.06	0.14	0.12	0.06
Valine	0.35	0.71	0.59	0.27
Dispensable amino acids				
Alanine	0.53	0.71	0.59	0.44
Aspartic acid	0.58	0.97	0.81	0.63
Cystine	0.16	0.29	0.24	0.15
Glutamic acid	1.26	2.21	1.84	2.36
Glycine	0.29	0.61	0.51	0.26
Proline	0.65	0.86	0.72	0.68
Serine	0.37	0.54	0.45	0.29
Tyrosine	0.24	0.53	0.44	0.31
Total amino acids	7.29	12.24	10.19	7.40

CN, corn; PB, plain bread meal; SB, sweet bread meal; BC, biscuit meal. All values are expressed on an as-fed basis.

**Table 2:** Composition of experimental diets ^1^.

Items, %	CND	PBD	SBD	BCD	N-Free
Ingredients					
Starch	27.35	27.35	27.35	27.35	68.00
Corn	50.00	-	-	-	-
By-product	-	50.00	50.00	50.00	-
Sucrose	20.00	20.00	20.00	20.00	20.00
Dicalcium phosphate	0.80	0.80	0.80	0.80	1.50
Soybean oil	-	-	-	-	3.00
Cellulose	-	-	-	-	4.00
Potassium carbonate	-	-	-	-	0.40
Magnesium oxide	-	-	-	-	0.10
Limestone	0.75	0.75	0.75	0.75	1.90
Salt	0.40	0.40	0.40	0.40	0.40
Vit-Min mix ^1^	0.30	0.30	0.30	0.30	0.30
Chromium oxide	0.40	0.40	0.40	0.40	0.40
Total	100.00	100.00	100.00	100.00	100.00
Analyzed value					
Dry matter	90.89	91.38	91.74	92.53	91.88
Crude protein	4.78	6.58	6.38	5.26	0.28
Indispensable amino acid					
Arginine	0.19	0.24	0.21	0.14	0.01
Histidine	0.10	0.17	0.17	0.08	0.00
Isoleucine	0.15	0.21	0.19	0.12	0.01
Leucine	0.61	0.39	0.33	0.21	0.02
Lysine	0.13	0.23	0.20	0.12	0.01
Methionine	0.09	0.11	0.10	0.08	0.01
Phenylalanine	0.24	0.29	0.25	0.17	0.02
Threonine	0.18	0.23	0.20	0.13	0.01
Tryptophan	0.04	0.09	0.06	0.04	0.00
Valine	0.26	0.32	0.28	0.17	0.02
Dispensable amino acid					
Alanine	0.39	0.33	0.29	0.24	0.02
Aspartic acid	0.35	0.35	0.30	0.25	0.02
Cystine	0.09	0.13	0.12	0.09	0.01
Glutamic acid	0.88	0.89	0.73	0.94	0.01
Glycine	0.20	0.29	0.25	0.17	0.01
Proline	0.43	0.35	0.30	0.29	0.02
Serine	0.18	0.18	0.16	0.12	0.01
Tyrosine	0.16	0.23	0.20	0.16	0.01
Total Amino Acid	4.67	5.03	4.34	3.52	0.22

CND, corn-based control diet; PBD, plain bread meal diet; SBD, sweet bread meal diet; BCD, biscuit meal diet; N-free, nitrogen-free diet. ^1^ Vitamin A as retinyl acetate, 10,622 IU; vitamin D3 as cholecalciferol, 1660 IU; vitamin E as DL-α-tocopheryl acetate, 66 IU; vitamin K as menadione nicotinamide bisulfate, 1.40 mg; thiamin as thiamine mononitrate, 1.08 mg; riboflavin, 6.49 mg; pyridoxine as pyridoxine hydrochloride, 0.98 mg; vitamin B12, 0.03 mg; D-pantothenic acid as D-calcium pantothenate, 23.2 mg; niacin, 43.4 mg; folic acid, 1.56 mg; biotin, 0.44 mg; Cu, 20 mg as copper chloride; Fe, 123 mg as iron sulfate; I, 1.24 mg as ethylenediamine dihydriodide; Mn, 59.4 mg as manganese hydroxychloride; Se, 0.27 mg as sodium selenite and selenium yeast; and Zn, 124.7 mg as zinc hydroxychloride.

**Table 3:** Apparent ileal digestibility of crude protein and amino acids in bakery and biscuit byproducts fed to growing pigs.

Items, %	CN	PB	SB	BC	SEM ^1^	*p*-Value
Crude protein	61.91 ^b^	68.51 ^a^	67.05 ^a^	60.34 ^b^	0.43	<0.001
Indispensable amino acids						
Arginine	67.78 ^a^	67.26 ^a^	64.27 ^ab^	58.04 ^b^	2.17	0.020
Histidine	67.43 ^a^	67.56 ^a^	64.50 ^ab^	59.15 ^b^	1.97	0.014
Isoleucine	67.89 ^a^	67.36 ^ab^	64.29 ^ab^	58.30 ^b^	2.20	0.029
Leucine	67.45	67.18	63.92	58.09	2.46	0.081
Lysine	67.86	67.33	64.23	58.34	2.83	0.076
Methionine	68.01 ^a^	67.59 ^a^	64.73 ^ab^	61.96 ^b^	1.34	0.005
Phenylalanine	67.77 ^a^	67.36 ^a^	64.29 ^ab^	58.68 ^b^	2.04	0.020
Threonine	67.66 ^a^	67.43 ^a^	64.46 ^ab^	57.21 ^b^	2.38	0.023
Tryptophan	68.46	67.80	64.69	64.09	1.57	0.114
Valine	67.77 ^a^	67.30 ^ab^	64.23 ^ab^	55.93 ^b^	2.70	0.027
Total IAA	67.84 ^a^	67.44 ^a^	64.37 ^ab^	58.70 ^b^	2.06	0.022
Dispensable amino acids						
Alanine	67.50 ^a^	67.17 ^a^	64.41 ^ab^	59.83 ^b^	1.93	0.022
Aspartic acid	67.52 ^a^	67.26 ^a^	64.29 ^ab^	61.36 ^b^	1.36	0.006
Cystine	68.02 ^a^	67.59 ^a^	64.43 ^ab^	62.37 ^b^	1.23	0.022
Glutamic acid	67.30 ^a^	67.09 ^a^	63.63 ^b^	67.67 ^a^	0.70	0.013
Glycine	67.30	67.24	64.05	59.24	2.20	0.089
Proline	67.29	67.07	63.96	62.62	1.31	0.054
Serine	67.60 ^a^	67.33 ^a^	64.61 ^ab^	59.39 ^b^	1.89	0.012
Tyrosine	67.63 ^a^	67.46 ^a^	64.29 ^ab^	62.58 ^b^	1.18	0.014
Total DAA	67.45 ^a^	67.42 ^a^	64.39 ^ab^	61.84 ^b^	1.28	0.005
Total amino acids	67.67 ^a^	67.44 ^a^	64.39 ^ab^	60.11 ^b^	1.70	0.013

CN, corn; PB, plain bread meal; SB, sweet bread meal; BC, biscuit meal; IAA, indispensable amino acids; DAA, dispensable amino acids. ^1^ Standard error of the mean. ^a,b^ Mean values in the same row with different superscripts indicate significant differences (*p* < 0.05).

**Table 4:** Standardized ileal digestibility of crude protein and amino acids in bakery and biscuit byproducts fed to growing pigs.

Items, %	CN	PB	SB	BC	SEM ^1^	*p*-Value
Crude protein	67.03 ^b^	72.26 ^a^	70.93 ^a^	65.10 ^c^	0.43	<0.001
Indispensable amino acids						
Arginine	78.17	75.88	74.27	73.22	2.17	0.310
Histidine	67.49 ^a^	67.61 ^a^	64.56 ^ab^	59.23 ^b^	1.97	0.014
Isoleucine	82.39	77.59	76.08	76.75	2.20	0.163
Leucine	79.13 ^b^	85.55 ^ab^	85.52 ^ab^	93.06 ^a^	2.46	0.025
Lysine	84.44	77.39	75.82	76.73	2.83	0.145
Methionine	89.20	86.22	85.30	87.06	1.34	0.096
Phenylalanine	88.57	84.85	84.55	88.52	2.04	0.244
Threonine	81.14	78.63	77.29	77.36	2.38	0.552
Tryptophan	68.65	67.90	64.80	64.22	1.57	0.111
Valine	88.76	84.49	84.17	89.10	2.70	0.352
Total IAA	80.84	78.64	77.28	78.21	2.06	0.593
Dispensable amino acids						
Alanine	81.69	84.23	84.19	84.30	1.93	0.468
Aspartic acid	80.52	80.51	79.82	80.32	1.36	0.991
Cystine	89.07 ^a^	83.49 ^b^	82.21 ^b^	84.63 ^b^	1.23	0.005
Glutamic acid	70.45 ^ab^	70.41 ^ab^	67.60 ^b^	70.87 ^a^	0.70	0.037
Glycine	80.96	76.76	75.05	76.07	2.20	0.229
Proline	78.96	81.64	80.97	80.52	1.31	0.380
Serine	77.58	77.82	76.65	75.32	1.89	0.586
Tyrosine	82.34 ^a^	78.13 ^b^	76.53 ^b^	77.94 ^b^	1.18	0.013
Total DAA	80.23	79.26	78.06	78.70	1.28	0.393
Total Amino Acid	80.56	78.93	77.64	78.45	1.70	0.530

CN, corn; PB, plain bread meal; SB, sweet bread meal; BC, biscuit meal; IAA, indispensable amino acids; DAA, dispensable amino acids. Each SID value of CP and AAs was calculated by correcting the apparent ileal digestibility for basal endogenous losses. Basal endogenous losses were determined from the CP and AA concentrations in the nitrogen-free diet and in the ileal digesta of pigs fed the nitrogen-free diet, and were as follows (g/kg of DM intake): CP, 2.70; Arg, 0.23; His, <0.01; Ile, 0.24; Leu, 0.78; Lys, 0.25; Met, 0.22; Phe, 0.55; Thr, 0.28; Trp, <0.01; Val, 0.61. ^1^ Standard error of the mean. ^a,b,c^ Means in the same row with different superscripts show significant differences (*p* < 0.05).

**Table 5:** Concentrations of standardized ileal digestible amino acids in bakery and biscuit byproducts fed to growing pigs (%, as-fed basis) ^1,2^.

Items	CN	PB	SB	BC
Indispensable amino acid				
Arginine	0.27	0.53	0.43	0.23
Histidine	0.15	0.23	0.18	0.08
Isoleucine	0.21	0.45	0.36	0.18
Leucine	0.78	0.92	0.76	0.44
Lysine	0.18	0.45	0.36	0.14
Methionine	0.14	0.19	0.15	0.10
Phenylalanine	0.31	0.58	0.48	0.28
Threonine	0.22	0.39	0.32	0.15
Tryptophan	0.04	0.10	0.08	0.04
Valine	0.31	0.60	0.50	0.24
Dispensable amino acid				
Alanine	0.43	0.60	0.49	0.37
Aspartic acid	0.47	0.78	0.64	0.51
Cystine	0.14	0.24	0.20	0.13
Glutamic acid	0.89	1.56	1.24	1.67
Glycine	0.24	0.47	0.38	0.20
Proline	0.51	0.70	0.58	0.55
Serine	0.29	0.42	0.34	0.22
Tyrosine	0.20	0.41	0.34	0.24

^1^ Abbreviation: CN, corn; PB, plain bread meal; SB, sweet bread meal; BC, biscuit meal. ^2^ The concentration (%, as-fed) of standardized ileal digestible AA was calculated by multiplying the concentration of each AA (as-fed basis) by the SID of each AA.

## Data Availability

All data generated during this study are available upon reasonable request from the corresponding author.

## Abbreviations

The following abbreviations are used in this manuscript:

AID	Apparent ileal digestibility
SID	Standardized ileal digestibility
CP	Crude protein
AAs	Amino acids
BEL	Basal endogenous losses
PB	Plain bread meal
SB	Sweet bread meal
BC	Biscuit meal
CND	Corn-based control diet
PBD	Plain bread meal diet
SBD	Sweet bread meal diet
BCD	Biscuit meal diet
